# The ocean: a key Earth system regulator with boundaries

**DOI:** 10.1093/nsr/nwag551

**Published:** 2026-09-02

**Authors:** Sabine Mathesius, Jonas Kaiser, Malin Ödalen, Johan Rockström, Boris Sakschewski, Levke Caesar

**Affiliations:** Potsdam Institute for Climate Impact Research (PIK), Member of the Leibniz Association, Germany; Potsdam Institute for Climate Impact Research (PIK), Member of the Leibniz Association, Germany; Potsdam Institute for Climate Impact Research (PIK), Member of the Leibniz Association, Germany; Potsdam Institute for Climate Impact Research (PIK), Member of the Leibniz Association, Germany; Institute of Environmental Science and Geography, University of Potsdam, Germany; Stockholm Resilience Centre, Stockholm University, Sweden; Potsdam Institute for Climate Impact Research (PIK), Member of the Leibniz Association, Germany; Potsdam Institute for Climate Impact Research (PIK), Member of the Leibniz Association, Germany

## Abstract

By regulating Earth’s climate and buffering the impacts of anthropogenic pressures, the ocean plays a crucial stabilizing role in the Earth system yet, in doing so, it is itself undergoing profound changes. This Perspective outlines the role of the ocean in the Earth system and discusses arguments for incorporating additional ocean processes into the Planetary Boundaries framework.

The Planetary Boundaries framework seeks to identify and quantify processes that maintain the planet in a Holocene-like state [1,2]. Substantial deviations from Holocene conditions would expose life on Earth, including humanity, to conditions it is not adapted to, with potentially severe or uncertain consequences [1,2]. A central goal of the framework is to determine the levels of anthropogenic perturbation at which such conditions risk disrupting the biosphere and the Earth system, and to define the extent to which human activities remain within a ‘safe operating space’ [1,2]. Most Planetary Boundaries refer to terrestrial or atmospheric processes, whereas the ocean is only partially represented. In this Perspective, we outline the ocean’s role in the Earth system and show how ocean research can inform new control variables for the Planetary Boundaries framework.

The ocean is a central component of the Earth system, covering >70% of the planet’s surface, regulating global temperatures, influencing the atmospheric concentration of CO_2_ and other greenhouse gases, supplying atmospheric moisture that contributes to precipitation over land and containing the vast majority of the planet’s living space [3]. Life on Earth originated in the ocean billions of years ago and over time, photosynthesizing marine cyanobacteria elevated atmospheric oxygen to near-modern levels [4], thereby enabling the conditions for complex life on land. Today, land and ocean each contribute roughly half of global oxygen production, but most of this oxygen is respired again within terrestrial and marine systems, leading to virtually zero net oxygen flux between these reservoirs on biological timescales [5]. The contemporary ocean is undergoing rapid warming, acidification, oxygen loss and ecosystem degradation, driven by climate change and other human pressures [6], yet continues to play a key role in stabilizing the Earth system through large uptake of anthropogenic carbon and heat. This reflects a remarkable degree of biogeochemical and physical resilience, yet it also represents a response to a profound anthropogenic disequilibrium, an ongoing adjustment to human-driven perturbations.

The ocean’s capacity to absorb, store and redistribute heat over long distances is central to Earth’s regional and global climatic conditions to which contemporary ecosystems are adapted. Ocean circulation and vertical mixing transfer water and heat between the surface and deep ocean [3]. The thermohaline circulation interconnects basins globally, while wind-driven gyres and finer-scale mixing redistribute heat and nutrients within and across regions. Due to the ocean’s vast volume and long turnover time, combined with the high specific heat capacity of water, it acts as a highly effective sink for anthropogenic heat and has absorbed ∼90% of the warming related to recent climate change [7,8]. By moderating global warming, it provides terrestrial ecosystems with additional time to adapt, while marine ecosystems are experiencing increasing thermal stress, particularly due to the rising frequency and intensity of marine heatwaves [8,9]. In addition, ocean warming reduces the solubility of oxygen and CO₂, with reduced oxygen solubility being one of the main drivers of ocean deoxygenation [5]. Surface warming also increases the temperature gradient between surface and deeper waters, which enhances stratification and reduces oxygen supply to the deep ocean, further exacerbating deoxygenation [5,8]. The reduced exchange of water masses also limits the upward transport of nutrient-rich deep waters to the surface ocean, which can lower biological productivity [6].

Warming-induced changes in evaporation and precipitation over the ocean, and freshwater input from melting land and sea ice, alter seawater salinity, typically freshening low-salinity areas and increasing salinity in high-salinity regions [3,6]. Like warming, salinity changes affect seawater density and thereby stratification and circulation, resulting in regionally heterogeneous effects on heat and carbon uptake [3,6]. Enhanced surface-water evaporation and increasing atmospheric moisture also have major impacts on terrestrial systems, including intensified precipitation, higher risks of extreme rainfall and flooding and stronger tropical cyclones [3]. Ocean warming also affects coastal ecosystems and communities through sea level rise and may reduce marine food availability due to declining marine ecosystem health [6].

In addition to absorbing heat, the ocean has mitigated climate change by taking up 20%–30% of anthropogenic CO₂ [6]. This uptake occurs largely through the solubility pump, which refers to the dissolution of CO₂ in seawater and its transport into the deep ocean through ocean circulation and vertical mixing, effectively isolating the carbon from the atmosphere for many centuries [3]. Since CO₂ reacts with seawater to form carbonic acid, global sea surface acidity has increased by ∼30% since the Industrial Revolution [3], accompanied by adverse effects on species vulnerable to acidification [3,6]. Rising acidity is partly buffered by the seawater carbonate system. However, acidification increasingly depletes carbonate ions, resulting in a decrease in buffer capacity and ocean carbon uptake efficiency [10], i.e. the amount of CO₂ absorbed relative to the increase in atmospheric CO₂. Increasing stratification reduces carbon transport into the deep ocean, diminishing the air–sea CO₂ gradient relative to a well-mixed state, and thereby tending to reduce further ocean CO₂ uptake. Despite these weakening feedbacks, the solubility pump remains by far the primary mechanism for marine uptake of anthropogenic CO₂ [11].

Biological CO₂ uptake by the biological carbon pump and coastal vegetated ecosystems contributes to the ocean’s carbon sink and long-term carbon storage [11]. The biological carbon pump refers to the ocean’s biological processes that transport carbon from the surface ocean into deeper waters in the form of organic matter. While on decadal timescales far less effective than the solubility pump, the biological carbon pump can form a long-term carbon sink even under steady-state Earth system conditions because a small fraction of organic matter is buried in sediments and effectively removed from the atmosphere on geological timescales [3]. In shallow coastal waters, sinking organic matter reaches the sediments faster, leading to less remineralization in the water column compared with the open ocean, where sinking biomass is exposed longer to microbial activity and largely remineralized before reaching the sea-floor [11]. Coastal blue carbon ecosystems, such as sea-grass meadows and mangrove forests, complement the biological carbon pump by sequestering carbon at exceptionally high rates per unit area and providing substantial long-term blue carbon storage in vegetation biomass and coastal sediments [3]. Organic carbon not retained in coastal ecosystems can undergo lateral transport, resulting in either export to the ocean interior via the biological carbon pump or remineralization to inorganic carbon and subsequent transport via the solubility pump [11].

Marine ecosystems are increasingly exposed to multiple stressors, amplifying risks to biodiversity, resilience and ecosystem functioning. Ocean acidification increases the energetic cost of calcification and causes physiological stress in many marine organisms, while ocean warming leads to thermal stress and alters species distributions [6]. Excess nutrient runoff e.g. from overfertilization, drives coastal eutrophication, leading to oxygen depletion and degradation of coastal ecosystems [6]. Additional pressures arise from overfishing, overexploitation of habitat-forming species, seabed disturbance from bottom trawling and other human activities and pollution by novel entities like plastics. While direct impacts on the biological carbon pump remain uncertain, the degradation of coastal ecosystems such as mangroves, kelp forests and sea-grass meadows reduces their carbon sequestration capacity [6].

In the Planetary Boundaries framework, the only explicit representation of the ocean is the ocean acidification boundary, with its control variable—the variable used to monitor a Planetary Boundary—being the global mean surface seawater aragonite saturation state [1,2]. The aragonite saturation state reflects the availability of carbonate ions in seawater and has been chosen as a control variable to indicate increasingly challenging conditions for marine calcifying organisms, some of which are highly vulnerable to ocean acidification. Recent studies demonstrate that the assumptions underlying the acidification boundary threshold overestimated safe conditions [12] and that the Planetary Boundary for ocean acidification has been transgressed in recent years [12,13]. In addition to the biological impacts, the ocean acidification boundary reflects the ocean’s chemical uptake of CO₂, one of the strongest negative feedbacks in the Earth system to anthropogenic CO₂ emissions.

Several other Planetary Boundaries implicitly involve marine processes. The Planetary Boundary of Biogeochemical Flows uses the phosphorous flux from freshwater systems into the ocean as control variable, with the threshold set to prevent widespread ocean anoxia [2]. One of the control variables for the Biosphere Integrity Planetary Boundary addresses genetic diversity, represented by the global extinction rate, including marine extinctions—yet relatively sparse data on marine species skew the metric toward terrestrial contributions. Finally, the Planetary Boundary of Novel Entities conceptually includes marine plastics and other synthetic substances that ultimately accumulate in the ocean [2].

Recent discussions within the ocean science and Planetary Boundaries research communities suggest the need for a more comprehensive and systematic inclusion of marine processes [13–15]. Proposed extensions of the framework are evaluated against three key criteria for control variables. First, there should be evidence that the process plays a significant role in maintaining Earth system stability and supporting Holocene-like conditions. Second, there should be evidence that a perturbation of this process could drive large-scale or potentially irreversible changes, either abruptly via tipping points or gradually through sustained long-term trends. Third, the proposed control variable should be reliably quantifiable over time, ideally through both observational and model data.

Many of the processes illustrated in Fig. 1 are currently being evaluated for their suitability as indicators of physical, chemical or biological dimensions of global ocean change with the potential to influence the Earth system as a whole. Key processes under review, along with exemplary candidate control variables, include the following:

- ocean warming (e.g. sea surface temperature, upper-ocean heat content);
- ocean deoxygenation (e.g. dissolved oxygen concentration, volume of hypoxic waters);
- change in functional marine biosphere integrity (e.g. biological carbon sequestration, marine biomass).

**Figure 1.**
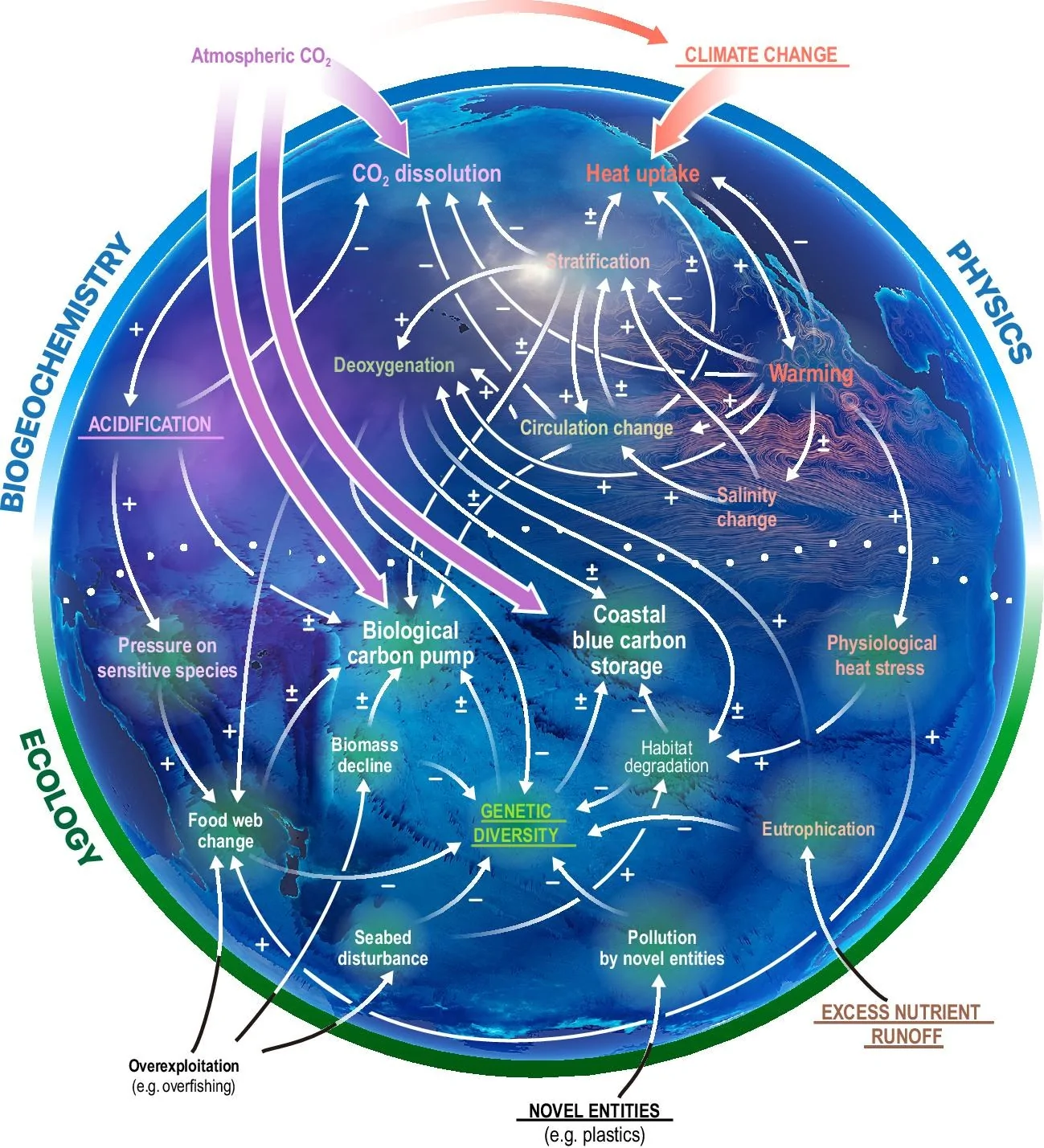
Key feedbacks between ocean physics, biogeochemistry and marine ecology in the Earth system under anthropogenic pressures. The figure illustrates how ocean uptake of CO₂ and heat drives physical, chemical and ecological changes, which in turn influence marine ecosystems and the ocean’s capacity to absorb further CO₂ and heat. Ocean warming, deoxygenation, acidification, overfishing, pollution and other anthropogenic pressures are placing severe stress on marine ecosystems. These disturbances not only threaten marine biodiversity but also undermine key Earth-system functions, including the ocean’s ability to absorb and store carbon. A ‘+’ symbol indicates a reinforcing process (positive feedback), whereas a ‘−’ symbol indicates a negative feedback. The ‘±’ symbols denote that changes can occur in both directions, with uncertainty regarding the net global effect. Capitalized and underscored words highlight terms already integrated into the Planetary Boundaries framework.

Ocean warming is under review because it reflects the moderation of climate change through ocean heat uptake, and changes in the ocean–atmosphere heat exchange could impact the Earth system at planetary scale. Furthermore, it is a major threat

to marine biosphere integrity, alongside ocean acidification and deoxygenation [6]. Arguments for integrating ocean deoxygenation into the Planetary Boundaries framework, proposed by the wider scientific community, include impacts on marine ecosystem functioning and potential long-term climate effects associated with nitrous oxide and methane emissions from anoxic waters [15]. The Biosphere Integrity Planetary Boundary currently does not include a component for marine functional integrity [2], warranting an assessment of whether a suitable marine counterpart to the terrestrial functional integrity control variable exists that meets the criteria for control variables.

In addition to considering the integration of new processes, existing marine components of the Planetary Boundaries framework are undergoing systematic revision. Ongoing work includes integrating the latest scientific evidence to reassess the ocean acidification boundary, potentially leading to an updated estimate of the safe operating space, as well as refining the genetic diversity control variable to more effectively capture marine genetic diversity.

Beyond its scientific value, the Planetary Boundaries framework also serves as a governance tool, providing a unifying language for assessing systemic environmental risk and guiding planetary stewardship. Improving its ocean representation will therefore not only enhance scientific accuracy but also strengthen the framework’s ability to inform marine governance. Different structural options exist for integrating new processes, for example, integration within closely related boundaries (e.g. ocean warming under the Climate Change Planetary Boundary), enhancing visibility across established policy domains or grouping of marine processes within an ocean-related boundary, emphasizing the multi-dimensional nature of ocean system change. Sustainable marine governance is essential for maintaining or restoring a safe and resilient ocean that sustains the rich diversity of marine life, while also serving as a key regulator of the Earth system. Incorporating the role of complex marine ecosystems into the Planetary Boundaries framework presents particular difficulties: their interconnectedness, three-dimensional dynamics and tight coupling between biological and physical processes posing a challenge for identifying thresholds and globally measurable indicators. Many critical functions, like biological carbon export, nutrient cycling and food web resilience, remain poorly observed, limiting our ability to assess resilience and feedbacks to the Earth system. Addressing these challenges will demand coordinated research linking ocean ecology, biogeochemistry and Earth system modelling. The Planetary Boundaries framework is designed to evolve with advancing science. By enhancing the representation of the ocean, drawing on the cumulative insights of the scientific community, it can more effectively capture planetary resilience amid intensifying human pressures.

## References

[1] Rockström J, Steffen W, Noone K et al. Ecol Soc 2009; 14: 32. 10.5751/ES-03180-140232

[2] Richardson K, Steffen W, Lucht W et al. Sci Adv 2023; 9: eadh2458. 10.1126/sciadv.adh2458 PMC10499318 37703365

[3] IPCC . Climate Change 2021: The Physical Science Basis: Contribution of Working Group I to the Sixth Assessment Report of the Intergovernmental Panel on Climate Change. Cambridge and New York: Cambridge University Press, 2023. 10.1017/9781009157896

[4] Lyons TW, Reinhard CT, Planavsky NJ. Nature 2014; 506: 307–15. 10.1038/nature13068 24553238

[5] Grégoire M, Oschlies A, Canfield D et al. Ocean oxygen: the role of the ocean in the oxygen we breathe and the threat of deoxygenation. In: Rodríguez Pérez A, Kellett P, Alexander B et al. (eds.). Future Science Brief. Ostend: European Marine Board, 2023. 10.5281/zenodo.7941157

[6] IPCC . Special Report on the Ocean and Cryosphere in a Changing Climate.Cambridge and New York: Cambridge University Press, 2019. 10.1017/9781009157964

[7] Von Schuckmann K, Moreira L, Cancet M et al. State Planet 2024; 4-osr8: 1–30. 10.5194/sp-4-osr8-1-2024

[8] Cheng L, von Schuckmann K, Abraham JP et al. Nat Rev Earth Environ 2022; 3: 776–94. 10.1038/s43017-022-00345-1

[9] Oliver ECJ, Donat MG, Burrows MT et al. Nat Commun 2018; 9: 1324. 10.1038/s41467-018-03732-9 PMC5893591 29636482

[10] Müller JD, Gruber N, Carter B et al. AGU Adv 2023; 4: e2023AV000875. 10.1029/2023AV000875

[11] Resplandy L, Lévy M, Bopp L. Sci Adv 2026; 12: eaed2480. 10.1126/sciadv.aed2480 PMC13189101 42160417

[12] Findlay HS, Feely RA, Jiang L et al. Glob Change Biol 2025; 31: e70238. 10.1111/gcb.70238 PMC12147064 40485607

[13] Sakschewski B, Caesar L, Andersen L et al. Planetary Health Check 2025: A Scientific Assessment of the State of the Planet. Potsdam Institute for Climate Impact Research (PIK), 2025. 10.48485/PIK.2025.017

[14] Nash KL, Cvitanovic C, Fulton EA et al. Nat Ecol Evol 2017; 1: 1625–34. 10.1038/s41559-017-0319-z 29066813

[15] Rose KC, Ferrer EM, Carpenter SR et al. Nat Ecol Evol 2024; 8: 1400–6. 10.1038/s41559-024-02448-y 39009849

